# Supplementary material for: Identification of photoperiod-induced specific miRNAs in the adrenal glands of Sunite sheep (Ovis aries)
Source: Front Vet Sci. 2022 Jul 22;9:888207. doi: 10.3389/fvets.2022.888207 (PMC9354845; doi:10.3389/fvets.2022.888207)
Supplement: Supplementary file 1 [file Data_Sheet_1.ZIP › Supplementary file/miRDeep2 file.zip!/miRDeep2 file/Novel_Structure/SP42A2/Novel_229.pdf]

Provisional ID	:	NC_019471.2_27387
Score total	:	2
Score for star read(s)	:	-1.3
Score for read counts	:	0
Score for mfe	:	1.7
Score for randfold	:	1.6
Score for cons. seed	:	
Total read count	:	96
Mature read count	:	96
Loop read count	:	0
Star read count	:	0

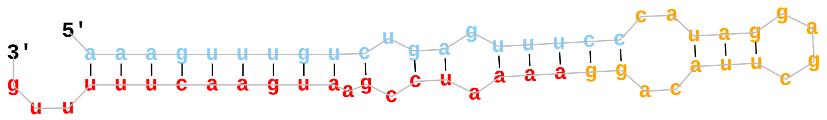

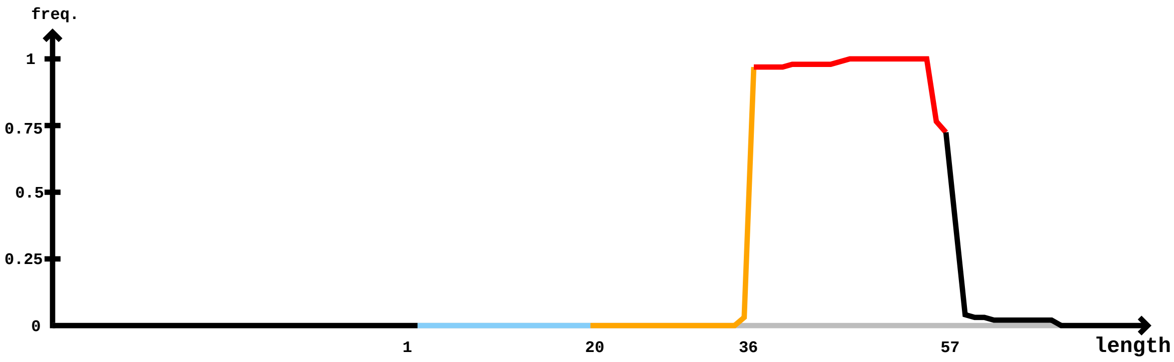

Star Mature

```
5'- cuuuuaaauuuaugcacauauguauuggguuggccaaaaguuugucugaguuucccauaggagcuuacaggaaaauccgaaugaacuuuuuggccaacccauauauuggccu  -3'   exp
    ............((.((.((((((.((((((((((((((((((((.((.(((((..(((....)))..))))).)).).)))))).))))))))))))))))))))))..   reads   mm   sample
    ......................................................................gaaaauccgaaugaacuuuuug....................   1   0   seq
    ......................................................................gaaaauccgaaugaacuuuuugg...................   2   0   seq
    .......................................................................aaaaucUgaaugaacuuuu......................   23   1   seq
    .......................................................................aaaauccgaaugaacuuuuu.....................   4   0   seq
    .......................................................................aaaaucAgaaugaacuuuuug....................   1   1   seq
    .......................................................................aaaauccgaaugaacuuuuug....................   3   0   seq
    .......................................................................aaaaucUgaaugaacuuuuug....................   57   1   seq
    .......................................................................aaaauccgaaugaacuuuuugg...................   3   0   seq
    .......................................................................aaaauccgaaugaacuuuuuggc..................   1   0   seq
    ...........................................................................uccgaaugaacuuuuuggUca................   1   1   seq
    ................................................................................augaacuuuuuggccaacccaGa.........   1   1   seq
    .................................................................................ugaacuuuuuggccaacccaGa.........   1   1   seq
```
